# The virus lesson: Teaching viral structure and quasi‐symmetry in mixed reality

**DOI:** 10.1002/pro.70570

**Published:** 2026-04-13

**Authors:** Adam Gardner, Fabien Cannac, Quentin Tallon, Arthur Olson, Ludovic Autin

**Affiliations:** ^1^ Department of Neurosciences University of California San Diego La Jolla California USA; ^2^ Department of Integrative Structural and Computational Biology The Scripps Research Institute La Jolla California USA

**Keywords:** augmented reality (AR), immersive, learning environment, mixed reality (MR), quasi‐equivalence, quasi‐symmetry, spatial colocation, virtual reality (VR), virus structure

## Abstract

Traditional approaches to teaching structural biology struggle to capture the dynamic, three‐dimensional nature of biomolecular structures. Viral capsids, which employ quasi‐symmetric arrangements, are especially difficult to conceptualize at the human scale. While physical models can illustrate geometry, they are costly, static, and disconnected from the digital domain. Recent advances in mixed reality (MR) offer an opportunity to overcome these limitations by combining immersion, interactivity, and collaboration. We present *Virus Lesson*, an MR application designed to teach viral quasi‐symmetry through shared immersive experiences. Developed in Unity and optimized for standalone MR headsets such as the Meta Quest 3/3S, the platform integrates multiplayer networking to allow students and instructors to co‐localize in the same virtual classroom. Activities include interactive exploration of virus size and composition, dissection of capsids to reveal encapsidated RNA, construction of quasi‐symmetric shells using Caspar‐Klug triangulation theory, and antibody–antigen recognition tasks. Educational design elements such as spatially separated “learning stations,” memory palace techniques, and scaffolded problem‐solving guide learners through increasingly complex concepts. Pilot demos with participants of all ages showed high engagement, improved comprehension of virus structure, quasi‐symmetry, and immunological interactions, and strong appreciation of collaborative learning in MR. The *Virus Lesson* demonstrates that MR can enhance understanding of challenging structural biology concepts while providing a scalable framework for interactive, human‐scale education in biomolecular sciences.

## 1 INTRODUCTION

Understanding biological structures requires not only knowledge of their components but also an appreciation of their three‐dimensional organization. This is particularly true for viruses, whose protective shells, called capsids, are built using geometric and quasi‐symmetric principles (Castón 2024; Johnson and Olson 2021; Johnson and Speir 1997). While textbooks, lectures, and laboratory exercises can explain the fundamentals (Castón 2024), they often fall short in conveying the spatial complexity and dynamic interactions of these assemblies. Because viral architectures exist far below the scale of human perception, students must rely on models and visualizations to grasp their intricacies.

One strategy is to use 3D‐printed models of viral capsids scaled up by several million times (Gardner and Olson 2016). Although these models effectively illustrate structure and symmetry, they are costly to produce and challenging to update with novel scientific data. Moreover, accurately representing mesoscale interactions requires a delicate balance of interactions (Davenport et al. 2017). As physical models lack digital integration, technologies such as augmented reality (AR) are required to depict dynamic properties, adding complexity (Eriksen et al. 2020; Gillet et al. 2005; Rodríguez et al. 2021).

Existing molecular viewers like *ChimeraX* (Goddard et al. 2018), *YASARA* (Ozvoldik et al. 2021), mol* (Sehnal et al. 2021), and *VMD* (Stone et al. 2016) now offer virtual reality (VR) extensions, but they are designed for expert users. They primarily emphasize free‐form exploration rather than structured pedagogy tailored to novice learners. In addition, several dedicated applications have emerged, such as UnityMol (Doutreligne et al. 2014; Laureanti et al. 2020), a Unity‐based VR platform featuring advanced molecular visualization and simulation. More recently, MolecularWebXR (Rodriguez et al. 2023) expanded this ecosystem with multi‐user WebXR environments where users on headsets, smartphones, or laptops can explore chemical and biological concepts together in shared 3D spaces. Other VR tools for microscopy and colocalization data also require prior biological knowledge (Theart et al. 2017) and are not focused on teaching concepts like quasi‐symmetry or capsid structure. Purpose‐built educational apps such as *LifeBrush* (Davison 2020) or *Peppy* (Doak et al. 2020) use VR to explore mesoscale environments or peptide structure, but they lack multiplayer features and a student–teacher dynamic. *CellexalVR* (Legetth et al. 2021) supports multi‐user analysis of single‐cell omics data, while *Cellverse* (Wang et al. 2022) introduces a student–teacher mode but limits the immersive component to a single user. Gamified biology experiences, including VR escape rooms (Christopoulos et al. 2023), demonstrate additional teaching potential. Furthermore, mixed reality (MR) with spatial colocalization has mostly been applied to anatomy and surgical training (Malik et al. 2023; Quero et al. 2019), or for topics outside of biology such as job training (Bödding et al. 2025). These applications have shown an increased efficacy in learning difficult medical concepts versus traditional methods (Malik et al. 2023).

Recent surveys on immersive molecular visualization, such as the STAR report by Kuťák et al. (2023), highlight the strong educational potential of immersive technologies while emphasizing the importance of instructional design and guided interaction for non‐expert learners. Together, these studies show clear educational benefits of MR, multiplayer colocalization, and guided instruction, yet no existing platform combines all three for teaching of viral structure, quasi‐symmetry, and capsid assembly.

To address this gap, we developed the *Virus Lesson*. Following David Goodsell's ethos from his PDB‐101 lessons, we used his 200th Molecule of the Month column (Goodsell 2016) “Quasi‐symmetry in Icosahedral Viruses” as an inspirational framework. The column summarizes the concept that many viruses are symmetrical assemblies composed of numerous small chemically identical subunits. It highlights nature's use of quasi‐symmetry to incorporate additional subunits for larger assemblies. Building upon these concepts, we designed the *Virus Lesson* with a series of activities to facilitate conceptual learning through interactive activity‐based critical thinking exercises. Delivering such embodied, spatial classroom experiences requires accessible visualization technology, which recent advances in MR hardware have now made both practical and affordable. Standalone headsets, such as the Meta Quest 3S, are available for around $300 and can run full MR applications without the need for external computers or sensors. By leveraging this recent hardware advance, combined with modern game engines such as Unity and multiplayer networking, the app enables instructors and students to co‐localize within a shared digital environment overlaid onto the physical space. Within this environment, viral capsids and their quasi‐symmetrical arrangements, Triangulation(T)‐Numbers (Caspar and Klug 1962), composition, and immunological interactions can be directly visualized and manipulated. Allowing for discussion and direct pedagogical collaboration between groups of students and their instructors.

In this paper, we describe the design and implementation of the *Virus Lesson*, each activity's rationale and educational take‐home message, and the deployment of the demo during several public events which highlight its broader potential for advancing science communication and outreach in structural biology.

## 2 APPROACH

Inspired by David Goodsell's use of natural interfaces, such as his paper model exercises, we found that emerging immersive technologies such as MR were appropriate to explore the structures and functions of biomolecules. These technologies present an immersive platform with a strong sense of presence, crucial for introducing structural biology education in a highly engaging and perceptually coherent way (Lønne et al. 2023). Such qualities are critical for visualizing and understanding complex molecular architectures. By combining these natural interactions in immersive 3D environments, MR can enhance both in‐person and remote teaching for science‐based, structure‐related subjects. As with many lessons on the PDB‐101 (Zardecki et al. 2022), exercises can be done in isolation. However, when experienced in a group setting, it not only bolsters learning, but also provides a framework for discourse and educational discovery at all levels (Kitchen et al. 2019; Van Der Meer et al. 2023).

Several benefits emerge from using purpose‐built MR, notably its spatial qualities and immersive nature. Virtual environments can be designed to promote focus while limiting distractions and multitasking (Liu et al. 2022). In the *Virus Lesson*, we leveraged this strength by creating a clean, clutter‐free workspace where each learning station only includes the required elements. As users progress through the objectives, unnecessary items are removed to maintain focus. Each station uses neutral colors, while learning targets are highlighted with distinct tones to guide attention. The physically separate stations also employ the “memory palace” method, or method of loci (Yates 2002), which enhances recall by linking concepts to specific spatial locations. Each station corresponds to a unique concept, engaging the brain's spatial organization of memory. Recent studies (Krokos et al. 2019) show that virtual memory palaces experienced through head‐mounted displays (HMDs) yield better recall than desktop experiences, suggesting that MR environments can strengthen memory retention and retrieval.

The *Virus Lesson* is built in Unity, which provides a robust graphics and rendering framework, physics engine, and access to third‐party plugins needed for the Meta Quest 3 and 3S. Plugins enable key features such as camera passthrough, spatial colocation, hand and avatar tracking, and multiplayer networking through Photon Fusion (v1.0). Developing multiplayer support on standalone headsets requires careful optimization of rendering and bandwidth. To keep synchronization efficient, we relied mainly on networked variables and used remote procedure calls only when necessary. We adopted a server and client model in which the first user, typically the teacher, acts as the server. All physics calculations run on the teacher's headset, and only the resulting object states are transmitted to other users.

Beginning with high resolution designs, we optimized game assets by reducing polygon count, texture complexity, and eliminating visual artifacts such as surface flickering caused by overlapping geometry, which can be distracting in immersive environments. To design the activities, two types of 3D geometries were modeled: furniture elements and biological structures. Furniture assets were modeled and optimized using Unity, Blender, Cinema 4D, and MeshLab (Cignoni et al. 2008). Biological structures, including proteins, capsids, and whole viruses, were derived from PDB entries (wwPDB consortium et al. 2019), CellPACK models (Johnson et al. 2014; Johnson et al. 2015), or YASARA PET world datasets (Ozvoldik et al. 2021). These molecular models were rendered in ePMV (Johnson et al. 2011) or Mol* (Sehnal et al. 2021) using coarse molecular surfaces to reduce polygon count while preserving the overall shape, and then exported as glTF files. Similar considerations apply to the physics engine, where we employ low‐resolution colliders, reduce solver iterations, utilize animations instead of simulations, and unify complex objects post‐simulation. In addition, activities are built around two simple and intuitive core actions: “grab,” mapped to the middle finger on the controller, and “interact,” mapped to the index finger. The *interact* action allows users to point with a hand‐directed laser and trigger behaviors such as pressing a button, moving a slider, painting, or initiating a grab, while the *grab* action is reserved exclusively for holding and moving objects. This minimal design ensures that users unfamiliar with MR controllers can reliably pick up and manipulate objects in the scene. More advanced controls were added through the thumb joystick. The joystick moves a grabbed object closer or farther along the Y‐axis and rotates it either around a default axis or around a marked axis defined by a “tacked” reference object. To further support learning, we incorporated phantom placeholders that guide users on where to position an object before an event occurs. This “drag, release, and snap” interaction was applied in multiple activities, including the central table tasks, the antibody (IgG) binding, the T‐number construction, and the symmetry tack placement. These strategies provide clear advantages and reduce confusion while maintaining interactivity and engagement.

## 3 RESULTS

In this section, we describe the different stations we designed, how they were structured, and our intended educational take‐home messages. Each activity can be completed independently and explored at different levels of detail, making the overall lesson flexible in both duration and target audience. We then provide a summary of the feedback collected from users during our various demonstration activities.

**FIGURE 1:**
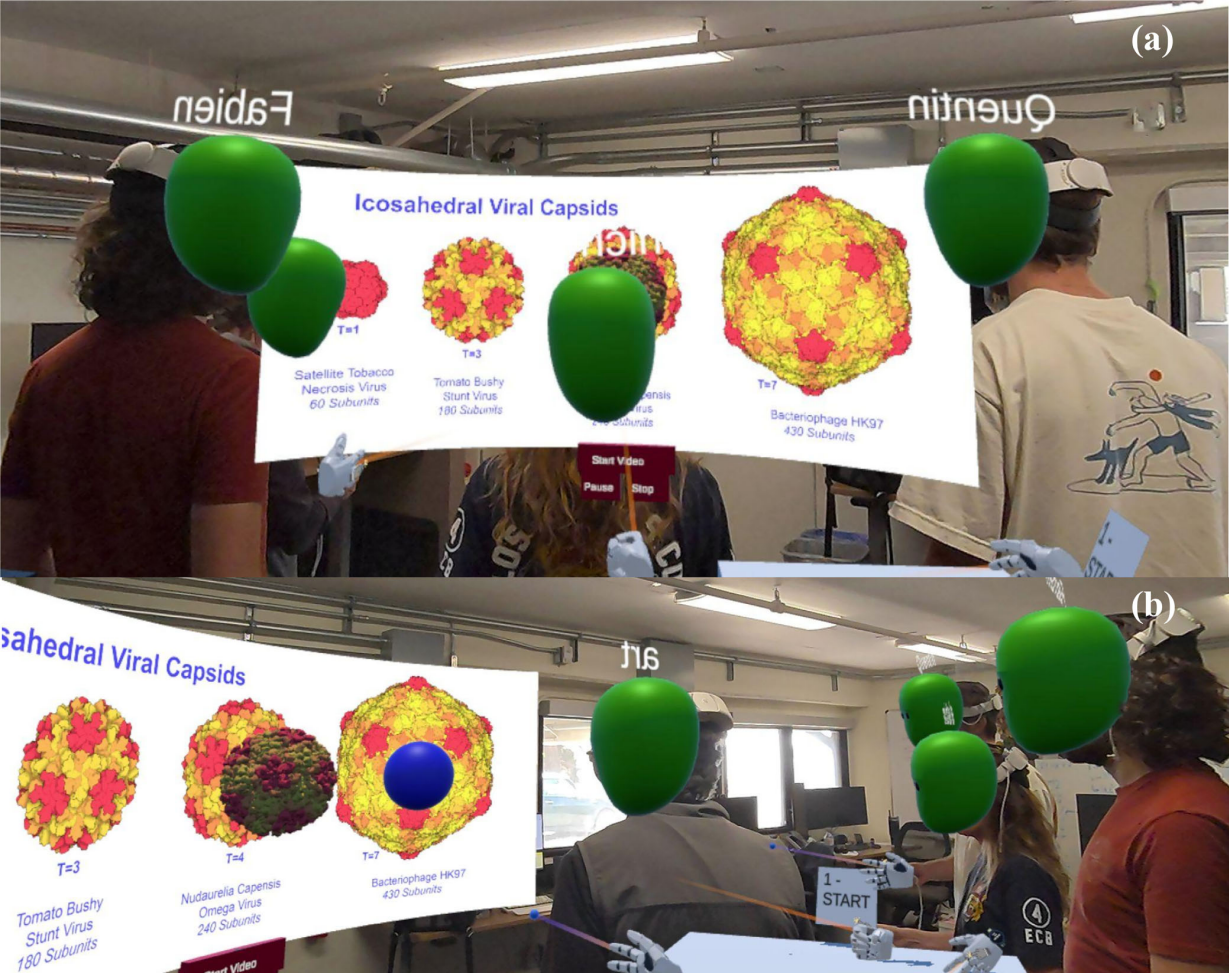
(a) Billboard of 4 iconic viruses for which participants can spawn. (b) 3D draggable representation.

In Figure 1, a billboard showing four viruses introduces the concept of size variation and shape similarities and is directly taken from the 200th Molecule of the month (Goodsell 2016). It comes to life when the students point at any of the viruses, producing a 3D model from the image. Students can grab and pull the model into their hands and examine it in detail, exploring how viruses are made of identical building blocks arranged in a regular pattern. By comparing different viruses, they discover that the number of building blocks increases with size. The billboard can become a video screen introducing and walking through the activities available in the app.

**Educational take‐home**: In this first phase of the lesson, users familiarize themselves with the MR environment, in which the experience is going to be located, and the basic interaction possibilities provided by the virtual world. Providing bounded physical spaces (i.e., the room) enables users to localize landmarks, facilitating navigation throughout the experience.

**FIGURE 2:**
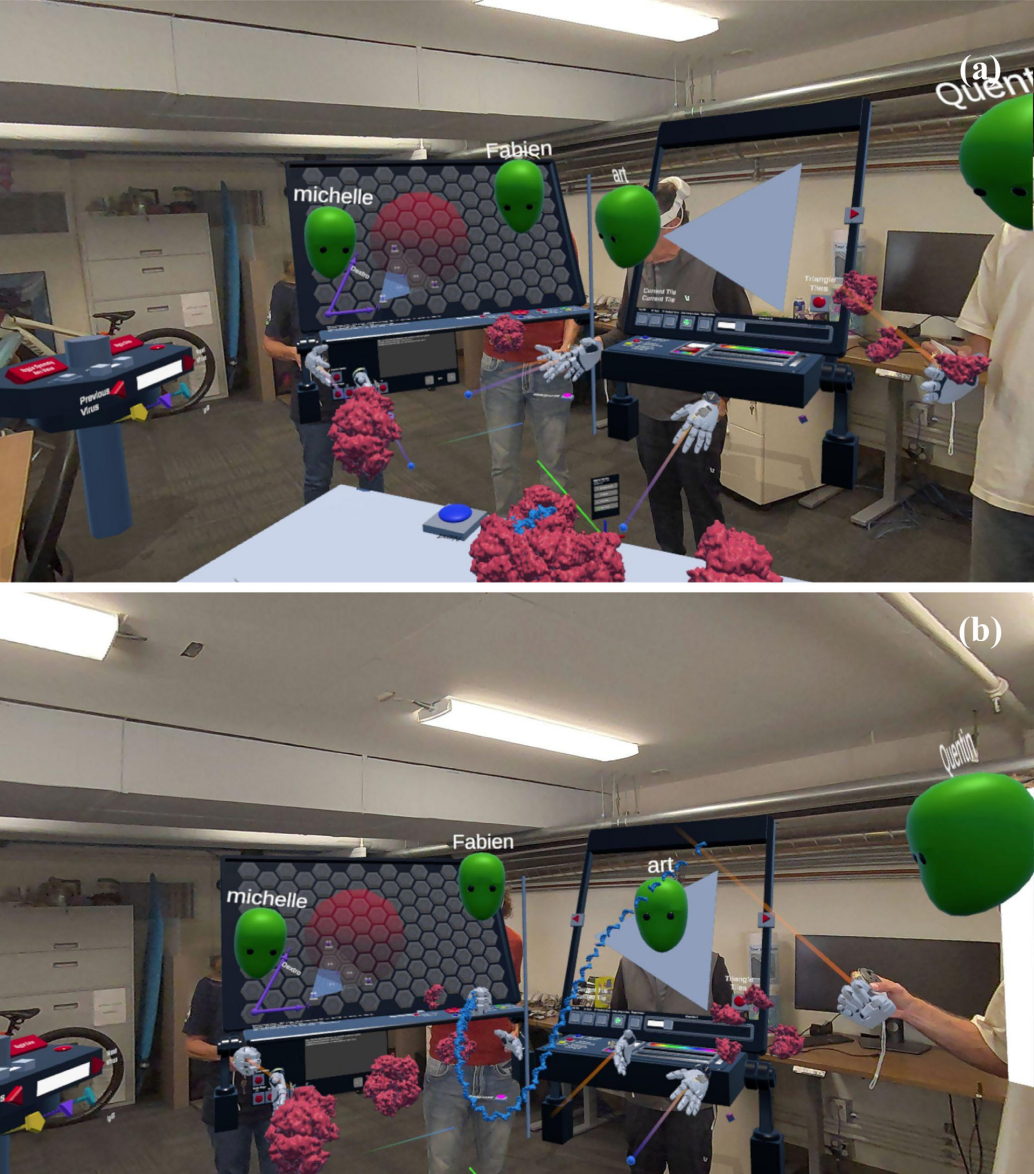
(a) A model of the Satellite Tobacco Necrosis Virus (STNV, 2buk). (b) Participants can disassemble the virus and discover the RNA inside (1239 nucleotides).

With guidance from the teacher, students move to the central table, proceeding through a series of activities that explore the function, geometry, and symmetry of viral capsids (Figure 2). At the push of the *Start* button, a small virus appears. One student can then grab individual protein building blocks or pentameric sub‐assemblies to examine their structure and composition. Like traditional dissection labs, capsid subunits can be removed, and genomic RNA can be pulled out and examined. Demonstrating how the capsid encloses the genetic material and releases it upon disassembly exposes its essential role in the viral life cycle. Discussing genetic efficiency and the use of having identical copies of a single protein building block, first hypothesized by Crick and Watson (Crick and Watson 1956), can further emphasize viral capsid's structure and function.

This step was technically challenging, requiring visualizing and manipulating individual capsid proteins and a flexible RNA chain while preserving interactivity. The capsid was generated with **ePMV** (Johnson et al. 2011) in *Cinema4D*, along with a chain consisting of one‐turn (10‐base) RNA fragment units, each connected by joints and physics colliders similar to methods we used in cellPAINT2D (Gardner et al. 2018; Gardner et al. 2021). To enable real‐time interactivity, the genome length was shortened.

**Educational take‐home**: This is the first truly interactive experience that the student encounters. By having them interact with a concept with which they might already be familiar (RNA is a single‐stranded chain protected by a cage made of proteins), they relate conceptual topics directly with their representation. This is a major educational strength of MR, cementing the understanding of ideas through direct interaction.

**FIGURE 3:**
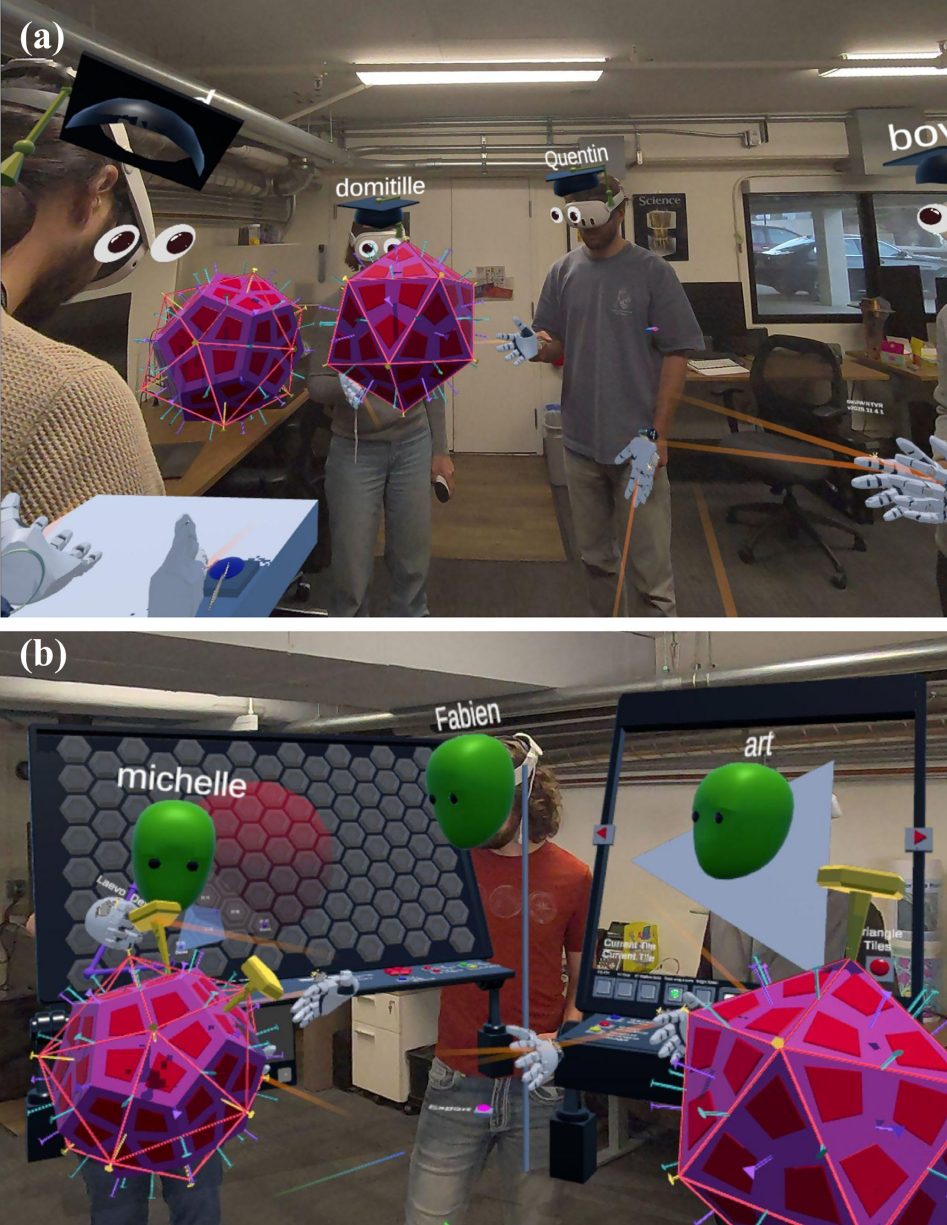
(a) Dodecahedron and Icosahedron presented to help participants identify unique subunits, the number of subunits, and symmetry axes. (b) Marker can be placed to identify symmetry axis.

Pushing the *Next* button produces two geometric solids: a dodecahedron and an icosahedron (Figure 3). This activity introduces students to the geometric concepts of regular polyhedra and symmetry. Identical kite‐shaped subunits are visible on both solids, and identifying the 12 pentagonal faces of the dodecahedron and the 20 triangular faces of the icosahedron demonstrates that each has 60 subunits. Both were easily generated in *Cinema 4D* using premade Platonic geometry. The symmetry axes, imported from Mol* (Sehnal et al. 2021), are displayed on the models and consistently color‐coded across activities, enabling students to recognize them at each stage. Comparing the two solids reveals that they share the same set of symmetry axes despite their different shapes. To reinforce these concepts, students can place “symmetry probes” (tacks) to highlight the 5‐, 3‐, and 2‐fold axes. Once probes are placed correctly, solids can be grabbed and spun interactively around the selected axes, giving students a direct, hands‐on experience of icosahedral symmetry (see below for more details on the tacks).

**Educational take‐home**: This activity provides exploration of geometric concepts, symmetry, and the relationship between subunits and icosahedral symmetry in the context of regular polyhedra. Learners gain an understanding of how the identical kite‐shaped subunits surround these solids, with the dodecahedron having 12 faces and the icosahedron having 20 faces. This also tests their recognition of symmetry axes in both shapes since the correct symmetry tack will only work when placed on their corresponding symmetry axis. The concept that icosahedral viruses can only have 60 symmetrically identical positions is made explicit.

**FIGURE 4:**
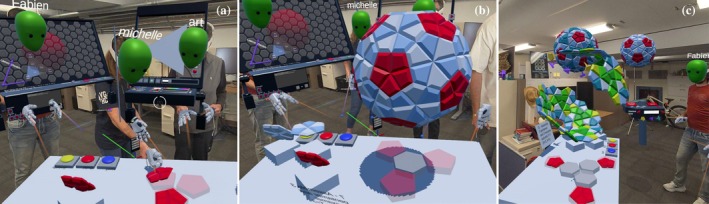
Capsid building table. (a) Build a T = 1 capsid model with pentamers. Assemble three on the template, with the center of the pentamer on the 5‐fold axis. (b) Build a T = 3 capsid model. As the name suggests, there will be 60X3 = 180 subunits in the virus. (c) Build a T = 7 capsid model with pentamers and hexamers. Hexamers hold 6 different chains/colors.

At the table the relationship between subunits and icosahedral symmetry is explored through a sequence of activities that gradually increase the number of subunits and, in turn, the size of the resulting capsid (Figure 4a–c). Using pentagonal and hexagonal tiles, students are introduced to the central concept of quasi‐symmetry: multiple copies of identical subunits are placed in different local environments at each of the 60 icosahedral positions, allowing construction of larger capsids than would be possible under strict local subunit icosahedral symmetry. To reinforce this concept, subunits in distinct environments are color‐coded. The number of unique symmetrical subunit positions defines the T‐number, with each subunit type exhibiting slightly different interactions based on its placement. Students access a dispenser that provides pentagonal and hexagonal tiles, colored to reflect local symmetry (two colors, three colors, or six colors) for the T = 1, T = 3, and T = 7 capsids explored at the table. After selecting a tile, students place it on a placeholder pattern representing one triangular face of the icosahedron. Once three pentagons and one or three hexagons are positioned, pressing the *Build* button triggers a real‐time assembly: starting from a single triangular face, the capsid grows as icosahedral symmetry operations are applied. The final structure is dynamically “relaxed” using Unity's physics system, with two springs per tile edge producing a visually compelling, virus‐like form. Activating “Solidify” freezes the structure, merging subunits into a single collider. This transformation is visible to all participants, reinforcing the shared experience. Symmetry tacks can then be placed to highlight relationships between different classes of subunits.

**Educational take‐home**: This activity fosters understanding of geometric concepts, symmetry, pattern recognition, and the relationship between subunits and capsid structures. It introduces the concept of quasi‐symmetry and T‐number. It encourages active exploration, problem‐solving, and the application of these concepts to real‐world examples in structural biology.

**FIGURE 5:**
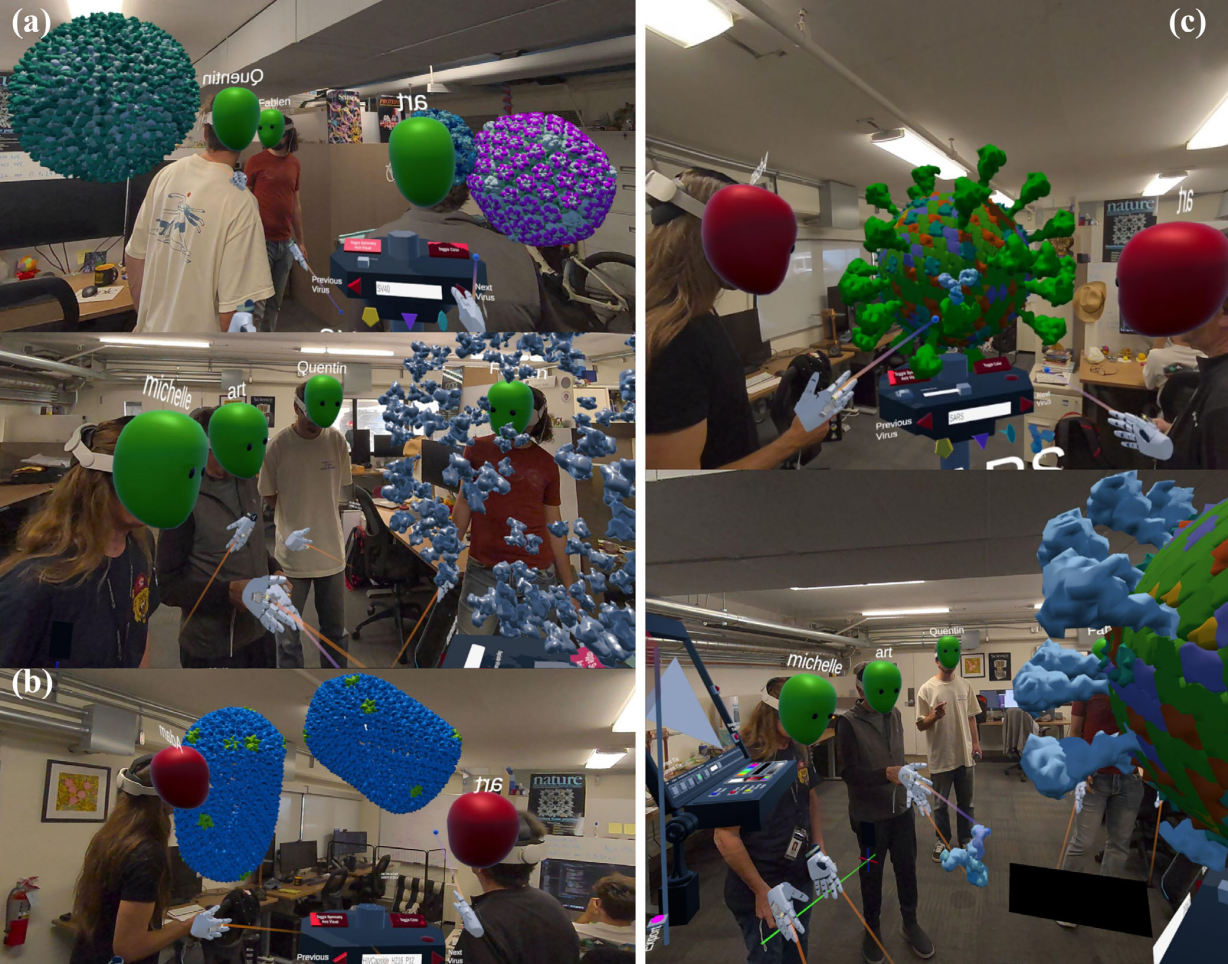
Virus explorer station. (a) The station can (1) display and spawn a dozen of iconic virus, (2) toggle the symmetry axes, (3) toggle the color, (4) explode the subunit along the symmetry axis. (b) The station can generate a new fullerene cone shape of HIV capsid. (c) The station let user explore mutation and IgG antibody binding illustrating immunology principle.

The next set of activities centers around the Virus Explorer station (Figure 5a), which is designed to show the wide variety of natural virus structures (see the full list in Table S1, Supporting Information). The collection includes numerous icosahedral viruses of different sizes and T‐numbers, as well as selected non‐icosahedral viruses [Influenza, HIV (Johnson et al. 2014), and SARS‐CoV‐2 (Ozvoldik et al. 2021)] that will be used in structural immunology and viral mutation activities. The focus here is for the user to identify the icosahedral symmetry axes in viral capsids using the symmetry probe tracks. The task can be performed with various levels of difficulty, with or without overlaid symmetry axes or subunit coloring. The icosahedral capsids can be expanded (exploded) successively into sub‐assemblies, and the concept and nature of quasi‐symmetry of chemically identical subunits can be demonstrated by examining sub‐assemblies of identical subunits that appear to be symmetric but may not fall on the true icosahedral symmetry axes and noticing obvious and subtle shape differences between individual protein subunits. Advanced examination of viruses like the Simian Virus 40 (Stehle et al. 1996) (PDB 1SVA) and the herpes simplex virus (Yuan et al. 2018) (PDB 1 5ZAP), which are exceptions to classical quasi‐symmetry, show how they fit into the same triangulation scheme.

**Educational take‐home**: At this station, students are introduced to actual viral diversity through the exploration of a selection of viruses with differing capsid arrangements. Using the interactive slider, students explode the viral capsids into their expanded sub‐assemblies. Naturally leading to the realization that, although these capsids are composed of identical subunits that appear to interact symmetrically, these individual subunits do not interact along the true icosahedral symmetry axes. This is a concept shared among the assortment of viruses. Through the hands‐on exploration of these capsid arrangements using symmetry tacks, students can mark major axes of symmetry and identify triangulation schemes. Thereby formulating and testing hypotheses that foster a deeper understanding about icosahedral symmetry, quasi‐symmetry, and exceptions like pseudo‐symmetry.

At the virus station the user is presented with various models of mostly static viral capsids. However there is one interactive non‐icosahedral capsid, the HIV capsid core particle, made with identical capsid protein molecules that form a fullerene (Schwerdtfeger et al. 2015) cone. As shown in Figure 5b, the user is allowed to create multiple versions of the capsid watching them grow (Hart 2009) and settle in different overall shapes despite each having exactly 12 pentamers and similar numbers of hexamers.

**Educational take‐home**: Students are presented with similar but different versions of HIV capsid. Allowing them to make observations of how protein capsids are organic but mathematically constrained and how the same subunit ingredients can yield different shapes and combinations.

The next lesson at this station integrates structural immunology concepts to broaden public understanding of immune responses and virus‐host interactions. At the virus station (Figure 5c), we have developed an activity centered around several membrane‐enveloped viruses and their surface proteins (S protein from SARS‐CoV‐2, NA/HA from influenza, and Env trimers from HIV). Students are introduced to rigid antibody models with both heavy and light chains targeted at specific antigen‐binding sites of the variant‐specific surface proteins. A color scheme reflects mutations in surface antigens, such that only the matching‐color antibody can recognize the corresponding antigen on the virus.

**Educational take‐home**: This activity integrates several concepts with which students may be partially familiar, including the distinction between antibody and antigens, their scale and manner of recognition, and the concept of virus mutations. We found that during the activity, the students would spontaneously be able to link these with concepts from recent news (such as variants of SARS‐COV2 viruses or HxNx influenza outbreaks) and roles of vaccines and boosters. Illustrating the powerful role visual and hands‐on demonstrations can have in explaining difficult topics.

**FIGURE 6:**
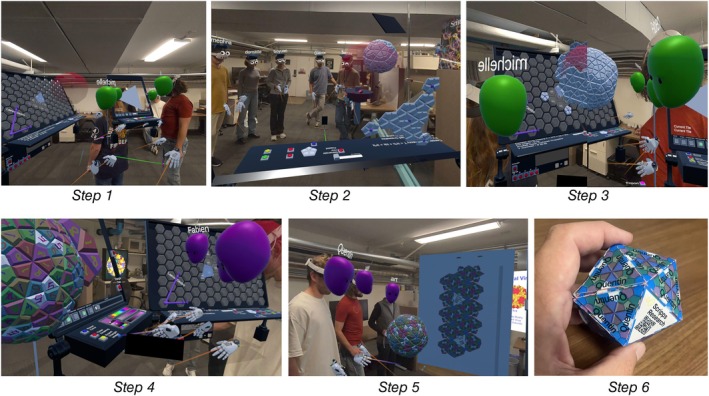
Caspar‐Klug T‐number generator station and interactive paint and fill station. Step 1: Select a desired T‐number using the interactive triangle. Step 2: Build Triangle button populates with the proper pentamers and hexamers. Step 3: Build Capsid button will start assembly of the tiles. Step 4: Participant can interactively draw directly on the tiles, as well as change their colors. Step 5: The flattened capsid can be exported to a 2D image for printing. Step 6: Printed and folded capsid generated by the participant.

The next activity station introduces the mathematical basis of capsid triangulation (T‐number) which determines the number of subunits in the complete capsid, and thus its size. Enabling students to generate viral capsids of any valid T‐number. Here, the concept is visualized on a board with a hexagonal net, beginning with the fact that hexagons can tile a flat surface. Curvature, and ultimately an enclosed capsid, can be introduced by replacing some hexagons with pentagons. A complete closure requires exactly 12 pentagons. This introduces the Caspar‐Klug theory (Caspar and Klug 1962) of icosahedral virus capsids and its mathematical expression, where the allowable T‐numbers are defined by triangles constrained to a hexagonal coordinate system. The interface to this net allows one to vary interactively the size of the triangle to define any T‐number particle (Figure 6, step 1). Once defined, the student can activate the construction of that particular capsid structure with a chosen type of ideal subunit (Figure 6, step 2). Advanced concepts such as the chirality of some T‐numbered capsids can be explored and explained.

Here, methods for dynamically reducing the complexity of models were essential to provide acceptable performance. The building of the capsid (Figure 6, step 3) is physics based and uses dual springs between the edges of tiles. When the capsid is completely built, via succession of symmetrical operations, the springs bring all the tiles together and the final shape is formed. While the building process could have been hidden and a completed T‐number icosahedral capsid could have appeared, we instead use a visible, step‐by‐step assembly to give the structure a more engaging and lifelike quality. This step is the most computationally expensive, so we developed and provided the ability to convert this group of individual tiles to a unified capsid. The individual tiles cannot be moved individually anymore to free up the physics solver and recover a proper interactive frame rate.

**Educational take‐home**: This activity highlights how MR can promote a deeper understanding of complex concepts such as the Caspar‐Klug Theory (Caspar and Klug 1962) and its relevance in molecular structures tying together mathematical and biological concepts. By constructing their own T‐number particles, students internalize the geometric logic behind the Caspar‐Klug theory, seeing how the arrangement of hexagons and pentagons governs curvature, closure, and capsid size. The interactive build makes an abstract mathematical framework tangible and memorable.

The final activities emphasize creativity and gamification. Using the custom T‐number capsid they generated, students can color individual subunits or draw freely on the surface using selectable colors and pen sizes (Figure 6, step 4). Their artwork is automatically propagated across the full capsid through icosahedral symmetry, allowing them to create unique and visually coherent designs. This activity is intentionally placed at the end as a reward, giving students an open, expressive, and collaborative moment after the more structured tasks. Once satisfied with their design, students can export the unfolded net (Figure 6, step 5) and print it to create a foldable physical paper model of their capsid (Figure 6, step 6 and Figure S1). The printed net includes the student's name and a QR code that links to additional information.

**FIGURE 7:**
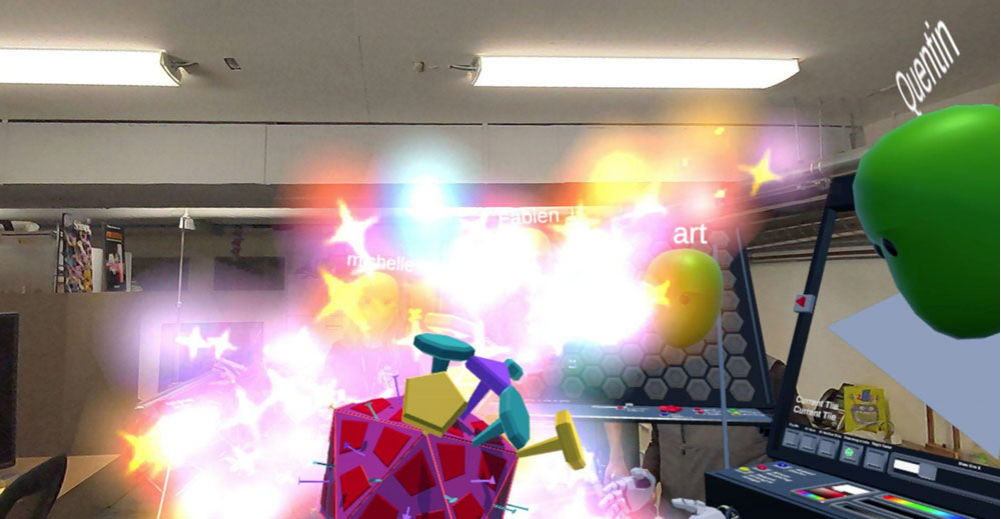
Symmetry axis retrieval game. This feature is available through all activities. Using 5‐, 3‐, and 2‐fold axis markers, students are tasked with identifying the corresponding symmetry axes on either existing viruses or newly built capsids. Providing audio feedback to reinforce correctly placed tacks and fireworks as a reward for completing the learning objective of recognizing all of the symmetrical axes in an icosahedral triangle.

This painted model can be used in a gamified activity to identify all symmetry axes in a single icosahedral triangle. If students successfully find the three 5‐fold axes, the three 2‐fold axes, and the central 3‐fold axis, congratulatory fireworks are displayed (Figure 7). The task can also be run as a friendly competition to see which student completes it the fastest.

**Educational take‐home**: This activity reinforces students' understanding of icosahedral symmetry by challenging them to locate and distinguish all major symmetry axes. It engages problem‐solving, attention to detail, and pattern recognition, while the fireworks provide positive reinforcement. Because symmetry tacks work on every viral structure in the lesson, students can increase the difficulty by exploring viruses with higher T‐numbers and more complex quasi‐symmetric patterns. Finally, exporting and assembling their own paper model creates a tangible bridge between the digital activity and a physical representation of viral geometry.

**Overall Key Educational take home**: Together, these activities cultivate careful observation, critical thinking, and an intuitive understanding of symmetry, quasi‐symmetry, and viral architecture. By progressing from guided exploration to open‐ended challenges, students repeatedly apply and test the concepts they have learned. The combination of intrinsic rewards (solving a spatial or structural puzzle) and extrinsic rewards (visual feedback, fireworks, competition) reinforces each stage of the lesson while building confidence in making, evaluating, and communicating scientific observations.

## 4 PUBLIC EVENTS AND GENERAL USER FEEDBACK

**FIGURE 8:**
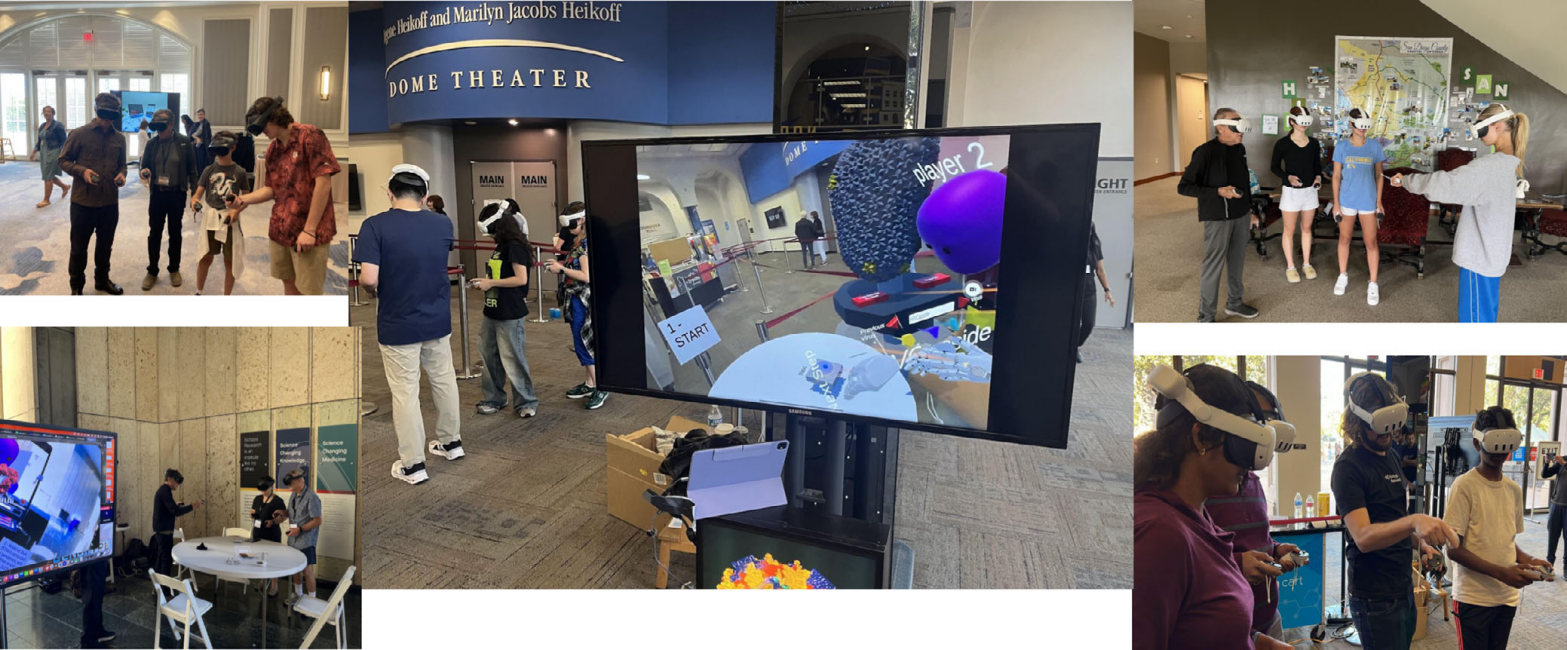
Pictures from our demo at various events including: the Scripps Family Retreat, Scripps Research public events, the Fleet Science Center, and a local high school illustrating the wide range of audiences we reached and our demo adaptability.

During development, we demonstrated the application in multiple settings (Figure 8): lab visits, graduate classes, middle and high schools, Scripps Research Public Front Row Lectures and other Institute events, Scripps Family Day, and the Fleet Science Museum. Feedback was collected through a 16‐question QR code survey (see Table S2).

Questionnaire responses from 36 participants from our most recent events at Scripps Research and the Fleet Science Museum showed consistently high engagement and enjoyment, with most users (94%) rating the experience 4 or 5 out of 5. Participants also reported improved understanding of viral 3D structure, quasi‐symmetry, viral diversity, antibody–virus interactions, and viral mutations. While these self‐reported gains suggest strong perceived educational value, they emphasize the need for a formal, controlled study to quantitatively assess learning outcomes. Across all demonstrations, simultaneous student groups ranged from two to six participants, covering a wide age range, although children under 10 could not participate because headsets were too large. Six simultaneous students appeared to be the upper limit for maintaining good performance and manageable group dynamics, particularly when most were young children. Children accompanied by a parent generally had a smoother and more focused experience.

As shown in Figure S2, ratings for enjoyment and excitement were high, and users evaluated comfort and performance positively, with only occasional reports of fatigue after longer sessions. The presence of instructors and peers within the MR space was also rated helpful, reinforcing the value of co‐localization and shared immersive learning. In open‐ended comments, participants described the experience as “awesome,” “super cool and innovative,” and “a very fun and interesting way to learn about viruses.” One participant specifically highlighted the activity where they could “pull out the RNA from the virus,” illustrating both engagement and perceived educational benefit.

## 5 DISCUSSION

Our pilot implementation of the *Virus Lesson* shows MR can provide an effective and engaging framework for teaching challenging structural biology concepts such as viral quasi‐symmetry. By integrating multiplayer networking, spatial scaffolding, and interactive manipulation of molecular structures, we observed that users not only enjoyed the experience but also reported improved comprehension of viral geometry, symmetry, diversity, and immunological interactions. These outcomes align with prior studies (Krokos et al. 2019), showing immersive environments enhance spatial reasoning and memory retention, extending benefits into the unfamiliar domain of structural virology. Importantly, the ability to co‐localize instructors and students in the same digital space highlights the potential of MR to support collaborative learning and broaden access to complex molecular topics.

During development of the Virus Lesson, advances in hardware enabled us to transition from VR to mixed reality (MR). We observed a noticeable reduction in cybersickness when users interacted through passthrough. VR cybersickness is generally attributed to sensory conflict, in particular a mismatch between visual input and vestibular cues, especially during visually simulated motion (Nürnberger et al. 2021). Reported symptoms can include nausea, dizziness, stomach awareness, fatigue, and sweating (Oh and Son 2022). In room scale MR, users walk physically and retain continuous visual feedback from the real environment, which likely reduces this sensory conflict. Controlled comparisons in simulation tasks suggest cybersickness can be lower or similar in MR than in matched VR conditions, and the effect appears sensitive to the amount of rendered graphics and visual complexity (Englebert et al. 2023; Kirollos and Merchant 2023). At the same time, passthrough is not inherently immune to discomfort, since camera‐based viewing can still induce sickness in some users, and geometry‐aware passthrough work supports the idea that visual distortions and depth or scale errors can contribute to symptoms (Chemaly et al. 2025; Santoso and Bailenson 2024; Stanney et al. 2020; Szentirmai et al. 2025). In practice, all of our users were able to remain in the headset in MR for more than 30 min without reporting discomfort.

The promise of the *Virus Lesson* lies in its adaptability and scalability. The app can serve as a general framework for teaching a wide range of structural biology topics beyond quasi‐symmetry, including protein folding, enzymatic function, ligand binding, DNA organization, and bioenergetics. Its modular design enables activities to be tailored to different educational levels, from introductory demonstrations to advanced explorations such as Caspar‐Klug theory (Caspar and Klug 1962). Furthermore, MR‐based platforms create opportunities for “mixed reality office hours,” in which vetted instructors could remotely guide students, expanding access to high‐quality structural biology education across geographic and institutional boundaries. These outreach possibilities are compelling for museums, science centers, and underserved communities.

## 6 LIMITATIONS

Several limitations remain. First, awareness and acceptance of MR benefits are still limited (Kirollos and Merchant 2023; Stromberga et al. 2021; Turhan and Gümüş 2022), and prior negative experiences with immersive technologies can reduce adoption. We therefore emphasize outreach in low‐pressure settings such as museums, science centers, and workshops to build familiarity and trust.

Second, performance constraints on standalone headsets affect both rendering and networking. Devices such as Meta Quest 3 and 3S impose limits on polygon count, draw calls, and compute throughput, and network performance degrades as more participants interact simultaneously. We mitigated these issues with low‐polygon assets, simplified rendering and textures, and a networking strategy that minimizes remote procedure calls and synchronized variables, while limiting typical sessions to two to six users. As standalone hardware improves, we expect these constraints to ease.

Third, onboarding remains a usability challenge because many users are unfamiliar with MR controls. We reduced interaction complexity to simple grab and trigger actions and used familiar metaphors such as wrist‐based interfaces, switches, and buttons. Ongoing iteration with user feedback remains essential to refine accessibility without reducing engagement.

## 7 FURTHER STUDY

While the *Virus Lesson* is a solid step towards a humanistic approach for teaching structural biology with MR and colocalized multiplayer, considerable growth remains possible. Expanding the lessons into other core areas of structural biology, traditionally difficult and abstract with other learning mediums, thus represents a straightforward next step.

With the recent rise of artificial intelligence (AI), a natural future direction is the integration of large language model‐based conversational agents that could support selected instructional roles, particularly for standalone use, while keeping human instruction preferable when available. AI may also enable richer AR workflows in which passthrough‐based recognition and tracking allow physical models, including 3D‐printed objects, to serve as tangible interfaces for virtual molecular content. In addition, future versions will expand annotation support beyond the current symmetry axis anchors to include labels and simple user‐placed annotations of structural features, which could further improve guided learning.

Finally, a more comprehensive evaluation framework is needed to compare the educational outcomes of mixed reality applications with those of traditional teaching methods. Although our preliminary surveys and early demonstrations indicate that immersive MR lessons may improve spatial reasoning, memory retention, and engagement, these instruments remain limited by low numbers and positive bias. A controlled user study will be essential to assess learning gains as well as *cybersickness and fatigue* (Kim et al. 2018). In parallel, improving our current survey by embedding it directly as a final in‐app MR activity coupled with a short quiz would provide more reliable, behavior‐based metrics. Because users are often reluctant to complete external questionnaires and tend to be overly positive, using in‐app interactions and performance indicators would yield more realistic insights into their experience.

The Virus Lesson shows that mixed reality can make complex structural biology concepts intuitive, collaborative, and engaging. As MR tools advance, this framework offers a promising path for accessible and scalable molecular education.

## AUTHOR CONTRIBUTIONS

**Adam Gardner:** Conceptualization; data curation; formal analysis; software; visualization; writing – original draft; writing – review and editing. **Fabien Cannac:** Data curation; writing – original draft; writing – review and editing. **Quentin Tallon:** Writing – review and editing; writing – original draft; data curation. **Arthur Olson:** Conceptualization; investigation; supervision; funding acquisition; writing – original draft; writing – review and editing; resources; project administration. **Ludovic Autin:** Conceptualization; investigation; funding acquisition; writing – original draft; methodology; writing – review and editing; software; formal analysis; supervision; visualization.

## CONFLICT OF INTEREST STATEMENT

The authors declare no conflicts of interest.

## Supporting information


**Table S1.** List of virus available at the virus column dispenser station.
**Figure S1.** Paper cut template for foldable capsid model.
**Table S2.** Survey question.
**Figure S2.** Survey answer summary.
**Video S1.** Teacher points of view.
**Video S2.** Student points of view.
**Video S2.** Spectator point of view.

## Data Availability

A standalone version of the application is available through the Meta Quest app store at this url: https://www.meta.com/experiences/the‐virus‐lesson/5389309677805355/. However, the multiplayer implementation remains only available upon request since large‐scale deployment is currently limited by the Photon Fusion ID system and requirements for precise room localization. Also, because multiplayer environments can carry the risk of harassment or disruptive behavior, we currently restrict group sessions to those facilitated by trained human instructors, while the app store version remains guided by text descriptions and video explanations. The source code is currently available on request.
