# Supplementary material for: The virus lesson: Teaching viral structure and quasi‐symmetry in mixed reality
Source: Protein Sci. 2026 Apr 13;35(5):e70570. doi: 10.1002/pro.70570 (PMC13073050; doi:10.1002/pro.70570)
Supplement: Supplementary file 1 — Table S1. List of virus available at the virus column dispenser station. Figure S1. Paper cut template for foldable capsid model. Table S2. Survey question. Figure S2. Survey answer summary. Video S1. Teacher points of view. Video S2. Student points of view. Video S2. Spectator point of view. [file PRO-35-e70570-s001.pdf]

Virus Name	PDB	DOI Citation
Norwalk virus	1IHM	B.V.V. Prasad, M.E. Hardy, T. Dokland, J. Bella, M.G. Rossmann, M.K. Estes, X-ray crystallographic structure of the Norwalk virus capsid (1999)
Yeast L-A virus	1M1C	J.R. Castón, J.L. Carrascosa, The L-A virus capsid (2001)
Nudaurelia Capensis Omega Virus	7ATA	D. Xu, et al., Structure of Nudaurelia capensis omega virus reveals ancient structural lineage of the double jelly roll superfamily (2021)
Bacteriophage HK97	1OHG	W.R. Wikoff, et al., Topologically linked protein rings in the bacteriophage HK97 capsid (2000)
Satellite Tobacco Necrosis Virus	2BUK	A.J. Olson, et al., Structure of satellite tobacco necrosis virus at 3.0 Å resolution (1983)
Canine Parvovirus (CPV)	2CAS	M. Luo, et al., Structure determination of canine parvovirus (1988)
Bacteriophage MS2	2MS2	P. Golmohammadi, et al., The crystal structure of bacteriophage MS2 coat protein (1993)
Tomato Bushy Stunt Virus (TBSV)	2TBV	J.D. Keown, et al., Tomato bushy stunt virus at 2.9 Å resolution (1978)
Human Rhinovirus 14 (HRV14)	4RHV	M.G. Rossmann, et al., Structure of a human rhinovirus complexed with its receptor molecule (1985)
Herpes Simplex Virus (HSV-2 B-capsid)	5ZAP	J. Huet, et al., Atomic structure of the herpes simplex virus type 2 B-capsid (2016)
Haloarcula californiae icosahedral virus	6H9C	A. Gil-Carton, et al., Archaeal virus HcIV-1: a giant among giants (2019)
Simian Virus 40 (SV40)	1SVA	T. Stehle, et al., Structure of simian virus 40 at 3.8-Å resolution (1996)
HIV Capsid	3J3Q	G. Zhao, et al., Mature HIV-1 capsid structure by cryo-electron microscopy and all-atom molecular dynamics (2013)
SARS-COV2	N/A	K. Ozvoldik et al., Assembly of Biomolecular Gigastructures and Visualization with the Vulkan Graphics API (2021)
HIV	N/A	G.T. Johnson et al., 3D molecular models of whole HIV-1 virions generated with cellPACK (2014)
Influenza	N/A	In house

Supplementary Table 1 - List of Virus available at the Virus column dispenser station. This table lists all virus structures accessible from the Virus Lesson dispenser station. For each virus, the corresponding PDB ID and citation are provided when available. Within the mixed reality environment, users can browse the full set of viruses, clone individual models, visualize their symmetry axes, and use an interactive slider to progressively "explode" each capsid along its symmetry directions to reveal subassemblies and highlight quasi-symmetric organization.

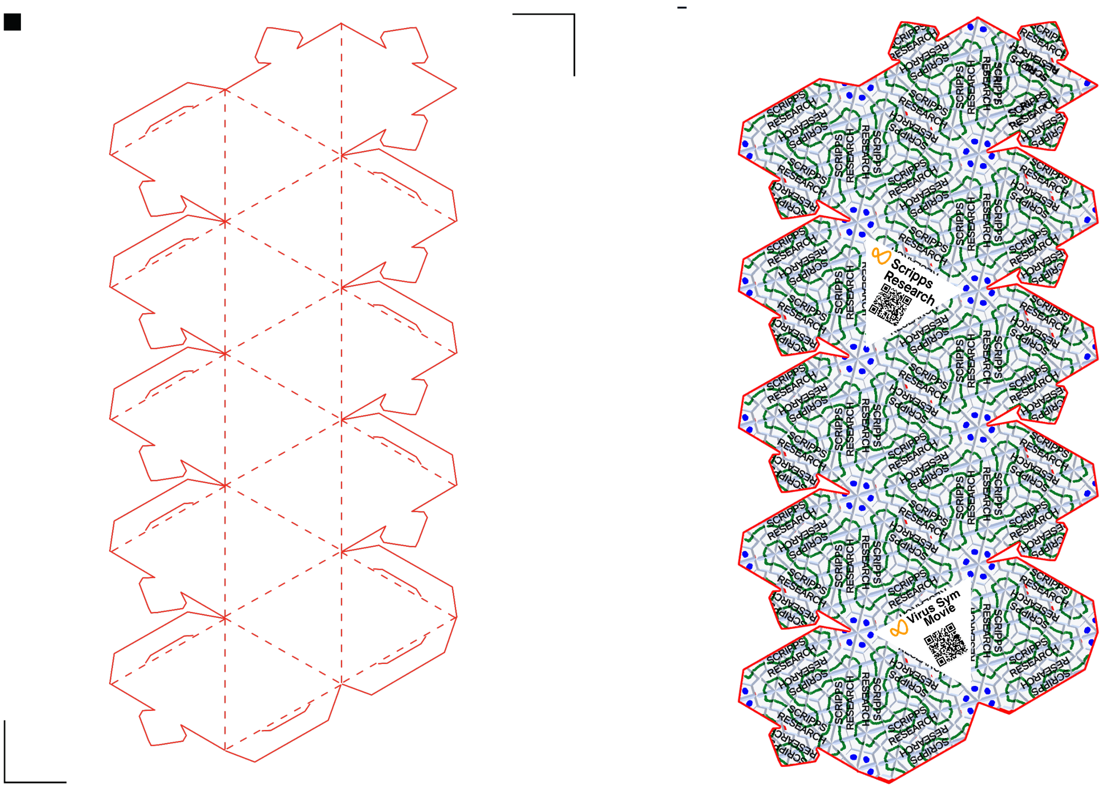

Supplementary Figure 1 – Paper Cut Template for Foldable Capsid Model
This figure shows, on the left, the template (Model-template2-transparent.png) used to generate a foldable paper model of the user-designed capsid. The layout includes cut lines (solid red), fold lines (dashed red), glue tabs, and orientation markers to assist with assembly. On the right is an example image produced by the app, showing the unfolded icosahedron with the user's pattern projected onto the net. QR codes printed on selected faces link to additional resources, including the Virus Lesson video demonstration and supporting information about capsid symmetry. The template is designed for cutting with a Silhouette machine and printing on cardstock, allowing users to physically reconstruct the icosahedral geometry explored within the mixed-reality application.

Q#	Question	Answer Options
1	Your age range	0–19 · 20–39 · 40–49 · 50–59 · 60–69 · >70
2	How enjoyable was the demo?	Not at all · 1 · 2 · 3 · 4 · 5 · Very much
3	Did the XR environment help you understand the 3D structure of viruses?	Not at all · 1 · 2 · 3 · 4 · 5 · Very much
4	Has your understanding of viral quasisymmetry improved?	Not at all · 1 · 2 · 3 · 4 · 5 · Very much
5	Has your grasp of virus diversity improved?	Not at all · 1 · 2 · 3 · 4 · 5 · Very much
6	Did the XR demo help you understand antibody–virus interactions?	Not at all · 1 · 2 · 3 · 4 · 5 · Very much
7	Did it improve your understanding of viral mutations?	Not at all · 1 · 2 · 3 · 4 · 5 · Very much
8	Did the XR environment provide an exciting way to learn?	Not at all · 1 · 2 · 3 · 4 · 5 · Very much
9	Did the XR environment feel like being in a room with the objects?	Not at all · 1 · 2 · 3 · 4 · 5 · Very much
10	Did having the teacher present in XR help you?	Not at all · 1 · 2 · 3 · 4 · 5 · Very much
11	Did having other students present in XR help you?	Not at all · 1 · 2 · 3 · 4 · 5 · Very much
	Motion sickness comfort	1 · 2 · 3 · 4 · 5
	Tiredness	1 · 2 · 3 · 4 · 5
	Performance	1 · 2 · 3 · 4 · 5
	Controllers	1 · 2 · 3 · 4 · 5
13	Additional feedback on the headset	Short answer
	Welcome table (virus opening)	Not relevant · Relevant · Very relevant · NA
	Building virus sizes at the table	Not relevant · Relevant · Very relevant · NA
	Virus gallery	Not relevant · Relevant · Very relevant · NA
	Honeycomb Caspar–Klug T-number generator	Not relevant · Relevant · Very relevant · NA
	Virus painting	Not relevant · Relevant · Very relevant · NA
	Symmetry-finding tasks	Not relevant · Relevant · Very relevant · NA
	Antibody–virus interactions	Not relevant · Relevant · Very relevant · NA
15	Overall feedback for the event	Short answer
16	Name (optional)	Short answer

Supplementary Table 2 - Survey Question
This table lists all questions included in the Virus Lesson feedback survey, along with the corresponding response options. The survey was administered to participants following the mixed reality demonstration to assess usability, comfort, and perceived educational impact of the application. All responses were collected anonymously.

A. Table of Average from 34 user ratings by category.

Main Category:	Sub Category:	Average Rating:	Standard Deviation
Learning	3D Structure of Viruses	4.71	±0.68
	Viral Quasisymmetry	4.47	±0.86
	Viral Diversity	4.52	±0.94
	Antibodies	4.47	±0.72
	Viral Mutation	4.35	±0.88
XR	Enjoyment	4.68	±0.77
	Excitement	4.55	±1.09
	Feeling of Presence	4.72	±0.68
Users	Teacher	4.79	±0.60
	Other Students	4.10	±1.09
Participant Comfort	Performance	4.27	±1.01
	Controllers	4.18	±1.01
	Motion Sickness	4.25	±1.05
	Feeling of Tiredness	3.91	±1.30
Key	5	4	3,2,1

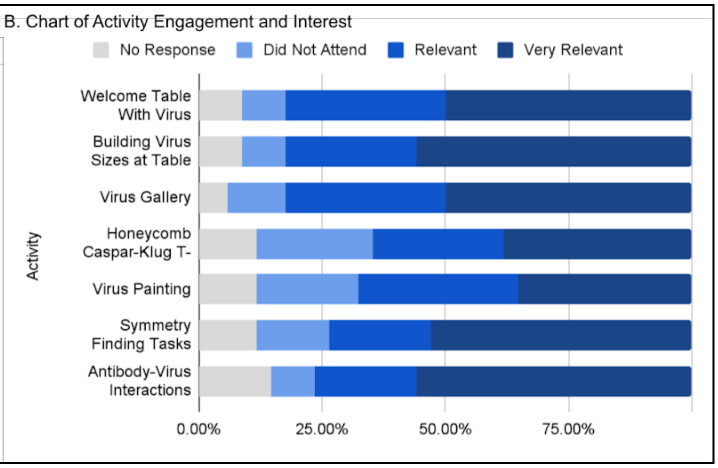

Supplementary Figure 2 - Survey Answer Summary

(**A**) Average ratings (1-5) for survey questions 2–12. This table summarizes participant responses to the survey questions assessing enjoyment (Q2), 3D structure understanding (Q3), quasisymmetry (Q4), viral diversity (Q5), antibody–virus interactions (Q6), viral mutations (Q7), excitement (Q8), sense of presence (Q9), usefulness of the teacher (Q10) and other students (Q11), and comfort metrics (Q12: performance, controllers, motion sickness, tiredness). Values represent the mean rating and standard deviation across 34 participants. (**B**) Engagement ratings for survey question 14. This stacked bar chart shows how participants rated each activity listed in Q14 (“Not relevant,” “Relevant,” “Very relevant,” or “Did not attend”). Activities include the welcome table virus opening, virus-size building table, virus gallery, Caspar-Klug T-number generator, virus painting, symmetry-finding tasks, and antibody-virus interaction activity.

Supplementary Video 1 – Teacher point of view
Supplementary Video 2 – Student point of view
Supplementary Video 3 – Spectator point of view
